# Alteration of gut microbiota in association with cholesterol gallstone formation in mice

**DOI:** 10.1186/s12876-017-0629-2

**Published:** 2017-06-09

**Authors:** Qihan Wang, Long Jiao, Chuanqi He, Haidong Sun, Qu Cai, Tianquan Han, Hai Hu

**Affiliations:** 1 Department of Surgery, Shanghai Institute of Digestive Surgery Ruijin Hospital, Shanghai Jiaotong University School of Medicine, 200025 Shanghai, China; 2 Institute of Gallstone Disease, Center of Gallbladder Disease, Shanghai East Hospital, Tongji University School of Medicine, #150 Jimo Road, Shanghai, 201200 China

**Keywords:** Gut microbiota, Cholesterol gallstone, 16S rRNA gene sequencing

## Abstract

**Background:**

The gut microbiome exerts extensive roles in metabolism of nutrients, pharmaceuticals, organic chemicals. Little has been known for the role of gut microbiota in regulating cholesterol and bile acids in association with gallstone formation. This study investigated the changes in the composition of gut microbiota in mice fed with lithogenic diet (LD).

**Methods:**

Adult male C57BL/6 J mice were fed with either lithogenic diet (1.25% cholesterol and 0.5% cholic acid) or chow diet as control for 56 days. The fecal microbiota were determined by 16S rRNA gene sequencing.

**Results:**

LD led to formation of cholesterol gallstone in mice. The richness and alpha diversity of gut microbial reduced in mice fed with LD. *Firmicutes* was significantly decreased from 59.71% under chow diet to 31.45% under LD, *P* < 0.01, as well as the ratio of *Firmicutes* to *Bacteroidetes*. Differences in gut microbiota composition were also observed at phylum, family and genus levels between the two groups.

**Conclusion:**

Our results suggested that gut microbiota dysbiosis might play an important role in the pathogenesis of cholesterol gallstone formation in mice.

**Electronic supplementary material:**

The online version of this article (doi:10.1186/s12876-017-0629-2) contains supplementary material, which is available to authorized users.

## Background

Gallstone disease is one of the most common gastrointestinal diseases in US and European countries [1, 2] with incidence around 10-15% among adults [3]. In Chinese Han population, its incidence increases close to western countries in recent years [4]. Almost 90% of the gallstones found at cholecystectomy were of cholesterol type [5]. Formation of cholesterol gallstone is a complex process through the interaction of genetic and environmental factors [6]. Supersaturation of biliary cholesterol due to either hyper-secretion of biliary cholesterol or decreased bile acids is believed to be prerequisite for the gallstone formation [7–10].

Gut microbiota play important roles in regulating the enterohepatic bile acid recycling process through modifying bile acid composition and pool size, and consequentially, influencing intestinal cholesterol absorption [11, 12]. Gut microbiota could profound change the physical characteristics of the bile acids [13–15]. Such regulation is also crucial for cholesterol metabolism because conversion of cholestrol into bile acids is a key step to get rid of excess cholesterol in the body [16]. Intestinal cholesterol absorption rate is much regulated by the hydrophobicity of bile acid composition as well [17]. Compared with primary bile acids, secondary bile acids have different critical micellar concentration and lower solubility in aqueous solution [18]. On the other hand, cholic acid (CA) and deoxycholic acid (DCA) have strong antimicrobial activity [19].

The gut microbiota may act as an “energy harvest organ” in digestion and metabolism of macromolecular nutrients in food as well as in synthesis of beneficial nutritional factors. They can stimulate intestine to establish an effective immune defense system, promote the renewal of intestinal mucosal cell and maintain the integrity of the intestinal tract [20]. Meanwhile, diet can have a strong impact on the species composition of the gut microbiota [21].

Gut micriobiota are reported to be associated with various disease especially metabolic disorder as obesity, diabetes [22]. However, it is still not clear on how the gut microbiota changes during the process of gallstone formation. In this study, we performed a large-scale sequences analysis of 16S rDNA in feces from gallstone susceptible C57BL/6 J mice fed with lithogenic diet. Our result suggested a role of gut microbiota dysbiosis in promoting gallstone formation.

## Methods

### Animal studies

Male C57BL/6 mice (age: 7–8 weeks) were purchased from Shanghai SLAC Laboratory Animal Co., Ltd. (Shanghai, China, license No. SCXK-HU 2012–0002). The mice were specific pathogen free (SPF) and were bred in a barrier environment at the Animal Care Facility of the Ruijin Hospital, Shanghai Jiaotong University School of Medicine on a 12-h light/12-h dark cycle in a controlled temperature (22.5 ± 2.5 °C) and humidity (50 ± 5%) environment. Two weeks after adaption to the environment, the mice were randomly assigned into two groups (8 mice/group) fed with either lithogenic diet (containing 1.25% cholesterol + 0.5% cholic acid, LD group) or chow diet (0.02% cholesterol, chow group) for 56 days. All the mice took water and designated food ad libitum during the experimental period. The experiment protocols were approved by the Ethical Committee at Ruijin Hospital, Shanghai Jiaotong University School of Medicine. All the procedures on animal experiment were reviewed and approved by the Animal Care Committee at Ruijin Hospital, Shanghai Jiaotong University School of Medicine.

On the day of sacrifice, the mice were euthanized by exsanguination after i.p. injection of chloral hydrate (350 mg/kg body weight). Twenty-four hour feces were collected from each mouse and stored at −80 °C until analysis.

### Genomic DNA Extraction and PCR Amplification

The E.Z.N.A. ® Stool DNA Kit (Omega Bio-tek, Norcross, USA) was used to isolate high-quality total microbial DNA from stool samples following the manual. The V4–V5 regions of the bacteria 16S ribosomal RNA gene were amplified by PCR. The forward primer used was 515 F: 5’-barcode-GTG CCA GCM GCC GCG G-3’, where the barcode is an eight-base sequence unique to each sample, and the reverse primer was 907R: 5’-CCG TCA ATT CMT TTR AGT TT-3’ [23]. PCR reactions were performed in triplicate. Each 20 μL reaction mixture contained 10 ng template DNA, 4 μL 5× FastPfu buffer, 2 μL 2.5 mM dNTPs, 0.8 μL of each primer (5 μM), and 0.4 μL FastPfu Polymerase. Reaction was performed at conditions including an initial step at 95 ° C for 2 min, followed by 25 cycles at 95 ° C for 30 s, 55 ° C for 30 s and 72 ° C for 30 s, and a final extension at 72 ° C for 5 min.

### Illumina miseq sequencing

Amplicons were purified with axyprep DNA gel extraction kit (Axygen Biosciences, Union City, Calif., USA) according to the manufacturer’s instructions. Purified amplicons were pooled in equimolar amounts and paired-end sequenced (2 × 250) on an Illumina miseq platform according to standard protocols. Raw data were deposited into the NCBI SRA (Sequence Read Archive) database.

### Processing of Sequencing Data using the QIIME software

Raw Illumina fasta files were demultiplexed, quality filtered, and analyzed using the QIIME software with the following criteria: (i) the 250-bp reads were truncated at any site of more than three sequential bases receiving a quality score < Q20, discarding the truncated reads that were shorter than 50 bp; (ii) exact barcode matching, with two nucleotide mismatches in primer matching; and (iii) only sequences that overlap longer than 10 bp were assembled according to their overlapping sequence. Reads that could not be assembled were discarded. OTUs (97% sequence similarity) were clustered using the UPARSE software (version7.1, http://drive5.com/uparse/), and chimeric sequences were identified and removed using the UCHIME program. The phylogenetic affiliation of each 16S rRNA gene sequence was analyzed using the Ribosomal Database Project (RDP) Classifier tool (version 11.1, http://rdp.cme.msu.edu/) against the SILVA (SSU115) 16S rRNA database (http://www.arb-silva.de/) using a confidence threshold of 70%. Once the number of sequence reads was homogenized between microcosms, alpha diversity was used to describe the microbial richness, diversity, and evenness within samples with four parameters: two richness estimators (Chao1 and the abundance-based cover-age estimator (ACE)) and two diversity indices (Shannon and Simpson indices). Jackknifed beta diversity analysis (between-sample diversity comparisons) was calculated using weighted and unweighted unifrac distances between samples, and principal coordinates were also computed to compress dimensionality into two-dimensional principal coordinate analysis (PCoA) plots. Observed species alpha rarefaction of filtered OTU tables was also performed to confirm that the sequence coverage was adequate to capture the species diversity observed in all samples.

### Statistics

Data are expressed as means ± SD. Differences between two groups were compared with *t*-test. Significance was defined as *P* < 0.05. Venn diagrams were used to represent shared and unique rare genera of microcosms among different groups. The threshold on the logarithmic Linear discriminant analysis (LDA) score for discriminative features was less than 2.0 (http://huttenhower.sph.harvard.edu/galaxy).

## Results

### LD decreased microbial richness and diversity

As expected, gallstones formed in all the mice fed with LD, but none in the chow group. LD increased plasma total cholesterol, LDL cholesterol levels. Liver weight, gallbladder volume and final body weight were also significantly higher in the LD group (Table 1).

**Table 1:** Effect of lithogenic diet on body weight, organ weights and plasma lipid levels

	Chow	LD
Initial body weight (g)	21.00 ± 0.76	21.73 ± 0.73
Final body weight (g)	21.48 ± 1.07	23.82 ± 1.24*
Liver weight (mg)	945.29 ± 160.66	1361.71 ± 179.72*
Gallbladder volume (μL)	13.29 ± 4.46	74.57 ± 29.38*
Plasma lipid		
TC (mmol/L)	2.92 ± 0.35	4.49 ± 1.16*
HDL (mmol/L)	2.35 ± 0.24	3.24 ± 0.80*
LDL (mmol/L)	0.09 ± 0.04	1.24 ± 0.52*

*TC* total cholesterol, *HDL* high-density lipoprotein, *LDL* low-density lipoprotein. ‘*’ represents *p* < 0.05

In the LD group, the observed OTUs, which represent the species numbers and richness of gut microbiota, were significant lower (226.14 ± 12.80 *vs* 263.00 ± 8.76, *P* < 0.01). The Shannon index decreased significantly in the LD group as well (3.42 ± 0.33 *vs* 4.32 ± 0.15, *P* < 0.01).

### LD remodeled the abundance of gut microbiota at different levels

**Fig. 1:**
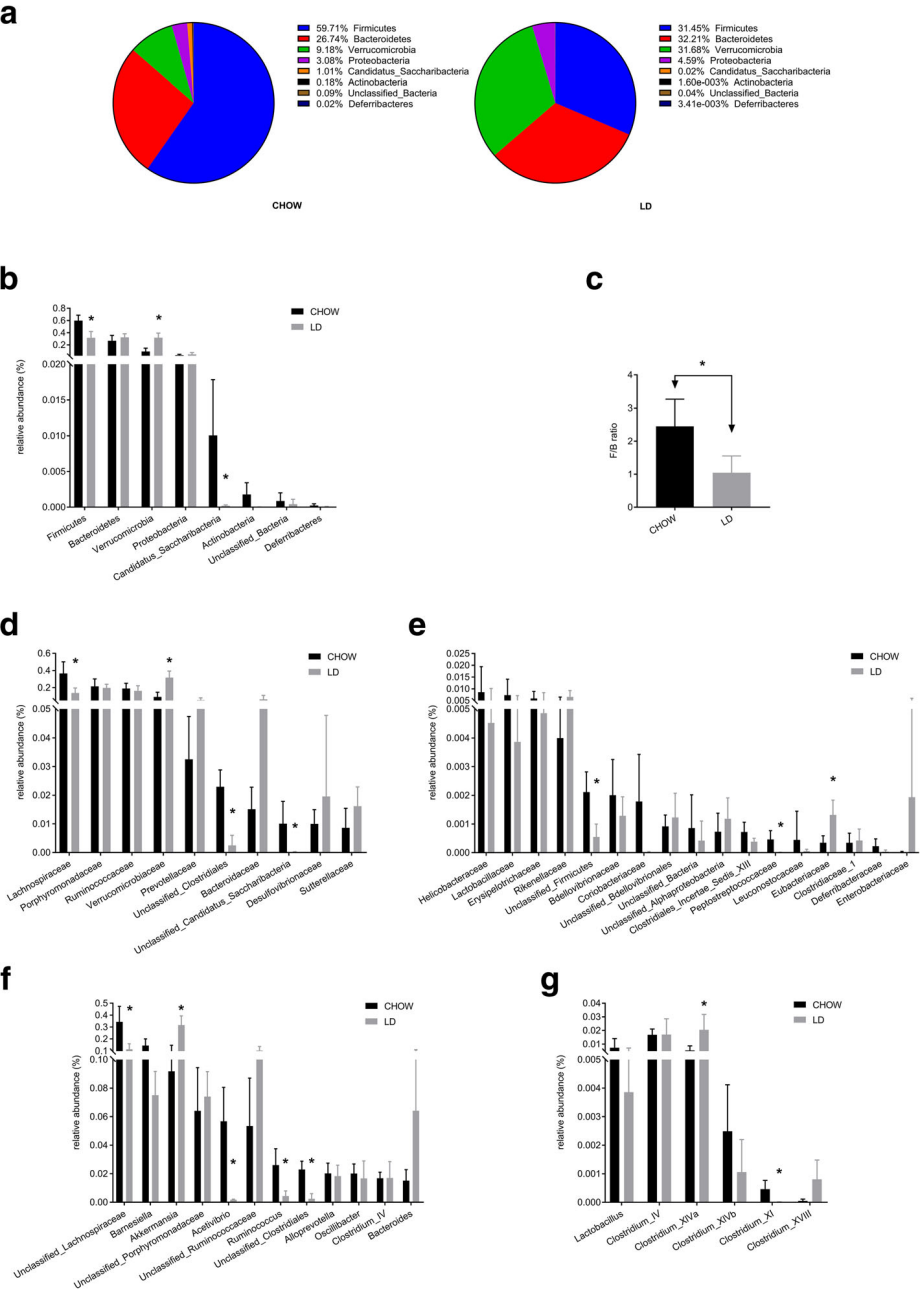
The abundance of gut microbiota at different levels. **a** Bacterial composition of the different communities at phylum level. **b** Relative abundance of the gut microbiota at phylum level. **c** The ratio between relative abundance of *Firmicutes* and *Bacteroidetes* (F/B). **d** Relative abundance of the top 10 families of gut microbiota. **e** Relative abundance of the rest families. **f** Relative abundance of the most abundance genera (>5% relative abundance). **g** Relative abundance of Lactobacillus and Clostridium. * was used to represent the significant difference (*p* < 0.05)

The relative abundance of *Firmicutes* and *Bacteroidetes* was >86% in the chow group, comprising majority of the gut microbiota (Fig. 1a and b). *Firmicutes* was the most prevalent phylum, comprised approximately 59.71% in chow group, but significantly decreased to 31.45% in LD group, *P* < 0.01, as well as *Candidatus Saccharibacteria* (Fig. 1a and b). In contrast, *Verrucomicrobia* significantly increased from 9.18% in the chow group to 31.68% in the LD group. Moreover, LD lowered the ratio of *Firmicutes* to *Bacteroidetes* (F/B) significantly (Fig. 1c), *P* < 0.01.

The family-level analysis illustrate that 10 families accounted for 96.32% and 97.14% of the total lineages in the LD and the chow groups, respectively (Fig. 1d and e). With the exception of unclassified subgroups, the LD led to higher *Verrucomicrobiaceae* abundance in comparison with chow diet, as well as *Eubacteriaceae*. On contrary, *Lachnospiraceae*, the most predominant family in the gut microbiota of the chow group, significantly decreased in the LD group, as well as *Peptostreptococcaceae*.

Fig. 1f showed the most abundant genera which had been found to be more than 5% relative abundance in the faeces. LD significantly increased *Akkermansia*. Meanwhile the relative abundance of *Acetivibrio*, *Ruminococcus* were remarkably reduced in the LD group compared with the chow group. Fig. 1g show the relative abundance of *Clostridium XlVa* were significantly higher in the LD group, *Clostridium XVIII* show the similar trend but insignificantly. While *Clostridium XI* significantly decreased in LD group as well as a tendency of less abundance of *Lactobacillus*.

**Fig. 2:**
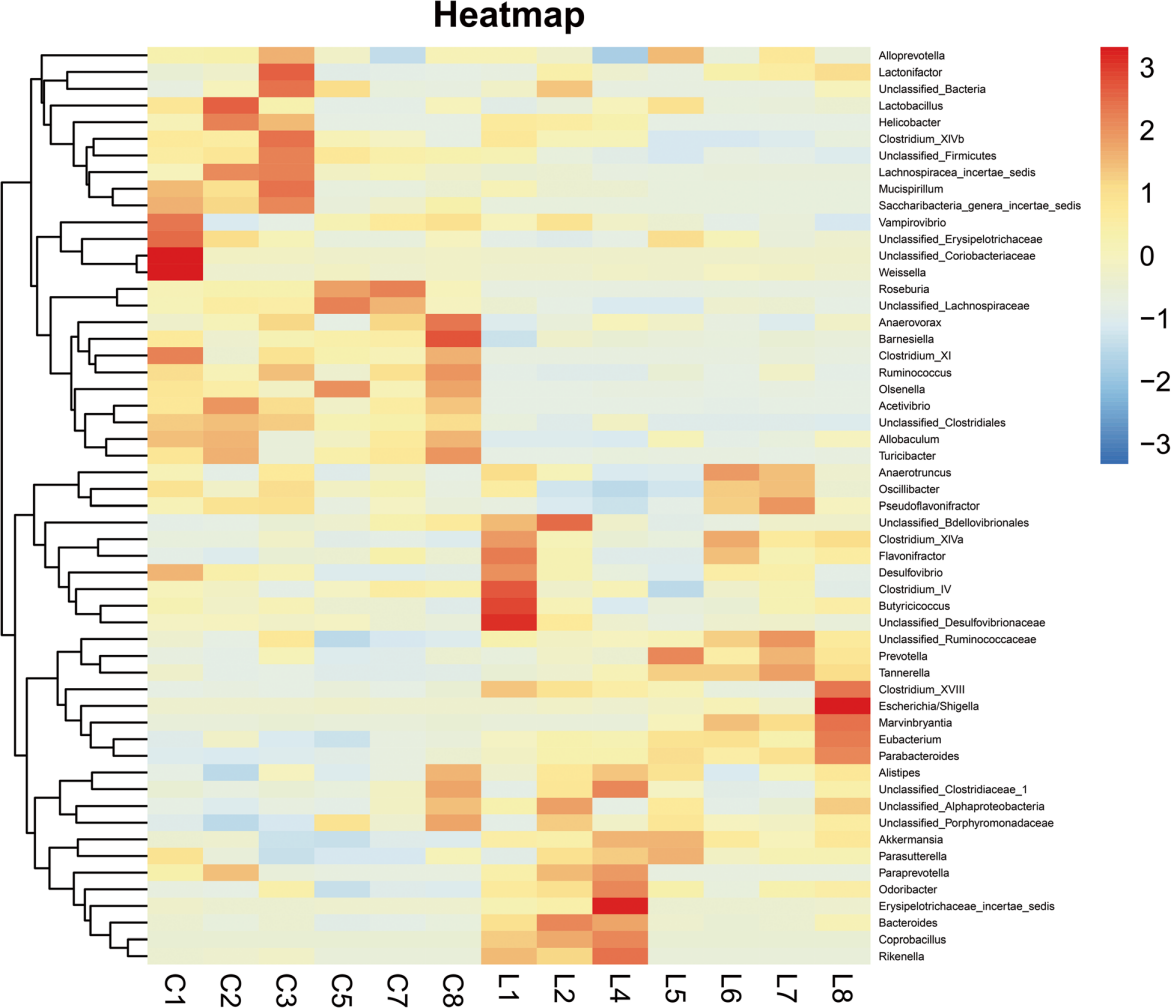
Heat-map diagram of the gut microbiota composition at genus level for all diet groups. The 55 genera that were shared by all samples tested (core microbiome) are displayed

The heatmap revealed a significant difference of relative abundance across the groups at the genus level (Fig. 2). It showed obvious increase of the genera *Akkermansia*, while the genera unclassified *Lachnospiraceae*, *Acetivibrio*, *Ruminococcus* and the genera unclassified *Clostridiales decreased*.

### Beta-diversity analysis of the microcosm composition

**Fig. 3:**
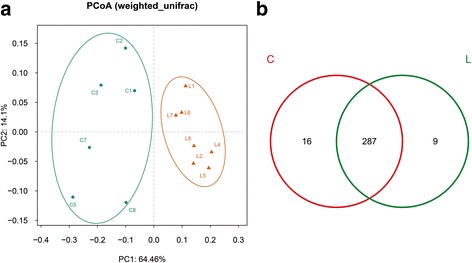
β-diversity and community similarity analysis of the microcosm composition. **a** Principal coordinates analysis (PCoA, weighted) of the microcosm composition. **b** Venn diagram representing shared and unique OTUs of the gut microbiome. Numbers in the diagram represent the number of OTUs in the different groups. There are 312 OTUs in all groups. C = chow group; L = LD group

The beta-diversity, which represented the extent of the similarity between microbial communities of two groups, was measured by Principal Coordinates Analysis (PCoA, weighted Unifrac Fig. 3a). The plot demonstrated significant divergence in the composition of gut microbiota between the LD and the chow groups.

### Community similarity and difference

The Venn diagram (Fig. 3b) demonstrate the shared and unique communities between the two groups. There were 287 OTUs shared by both groups, accounting for 91.99% of the total 312 OTUs in all groups. The chow group had 16 unique bacterial taxa, while the LD group had 9 (as listed in Additional file 1).

### LEfSe analysis of phylogenetic and taxonomic profiles

**Fig. 4:**
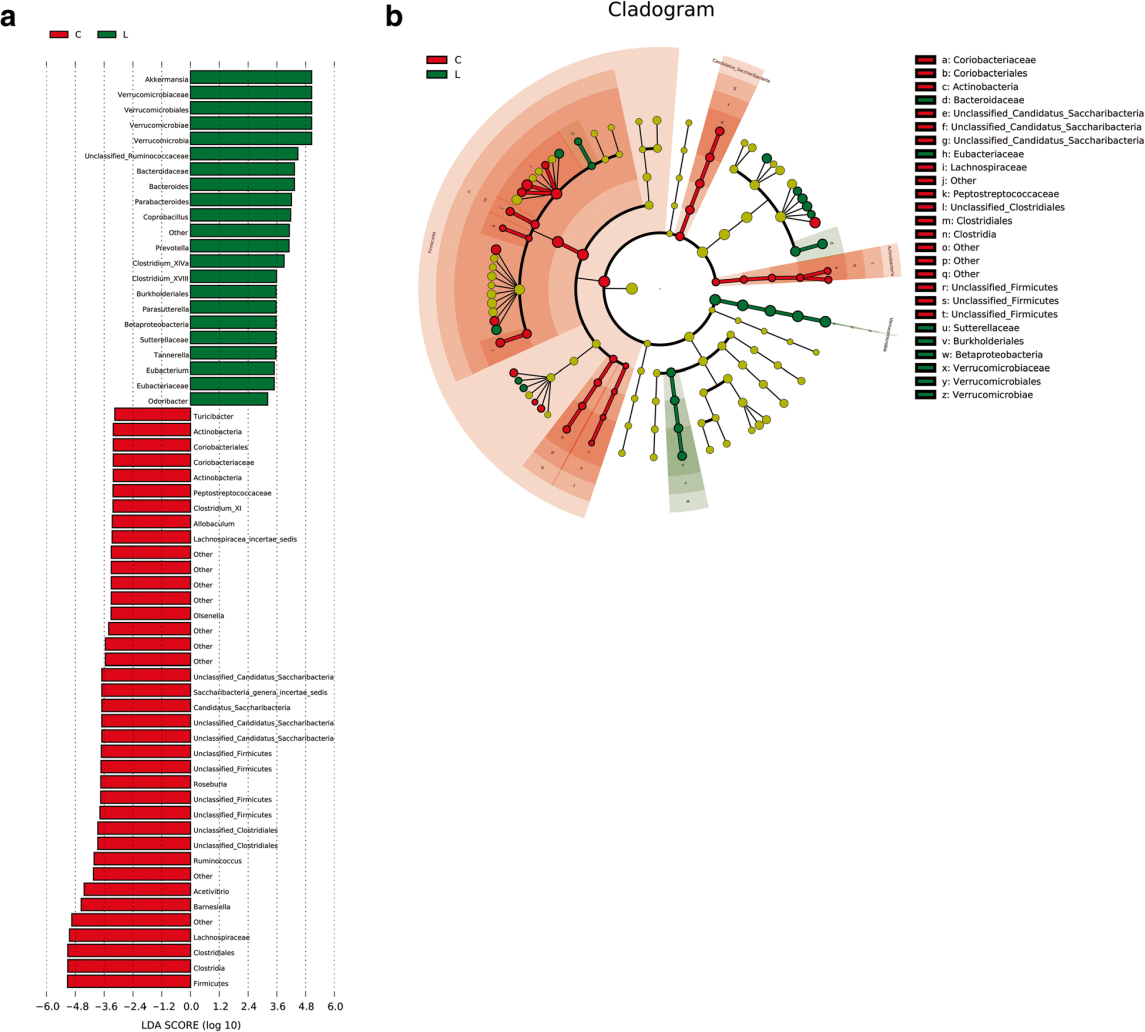
Different structures of gut microbiota in chow and LD group by LEfSE analysis. **a** Specific phylotypes of gut bacteria in response to lithogenic diet using LEfSe. The histogram shows the LDA scores computed for features at the OTU level. The lateral text shows the taxonomic profiles of all the OTUs, which were significantly different between the LD and the chow groups. **b** LEfSe cladogram in red for the taxa enriched in chow group and in *green* for the taxa enriched in LD group. The diameter of each circle is proportional to its abundance. C = chow group; L = LD group

The LDA effect size (LEfSe) analysis according to LDA scores shows that 60 OTUs were significantly different between the LD and the chow groups (Fig. 4a). The relative abundances of 22 OTUs were higher in the LD group. However, 38 OTUs were more abundant in the chow group.

Cladogram generated from LEfSe analysis showed the most differentially abundant taxa enriched in microbiota from mice in chow and LD groups. The LD group showed significant decrease in the *Firmicutes* and *Candidatus Saccharibacteria* phylum, as well as a more abundance of *Verrucomicrobia*, when compared with the chow group (Fig. 4b).

## Discussion

The present study showed that, in the mouse model of gallstone disease induced by lithogenic diet, the diversity of gut microbiota was altered. *Firmicutes* and the ratio of *Firmicutes* to *Bacteroidetes* all decreased. The gut microbiota was remodeling by LD at different levels. These results suggested that an important role of gut microbiota contributing to the formation of gallstone.

Although certain bacteria have been proposed to play a role in the pathogenesis of gallstone disease, few studies have ever investigated the changes of gut microbiota during the process of gallstone formation. In a previous study by Maurer et al. [24], they found that in gallstone-susceptible C57L/J mice, mono-infection of *Helicobacter bilis* or co-infection with *Helicobacter hepaticus* and *Helicobacter rodentium* led to significantly higher prevalence of cholesterol gallstone. This suggested certain strains of *Helicobacter* could promote gallstone formation. By sequencing the V4-V5 region of the 16S rRNA of bacteria, our results provided more evidences of changes in gut microbiota at the different levels in accompany with gallstone formation. Interestingly, alteration of indigenous gut microbiota by bacteria transferring has been shown to induce cholesterol gallstone formation in germ-free mice [25].

Gut microbiota affect the pathogenesis of gallstone disease through several mechanisms. Intestinal bacteria regulate bile acids metabolism through bile salt hydrolases (BSH) activity that de-conjugates bile acids and 7α-dehydroxylase activity that converts primary bile acids to secondary bile acids [14, 26, 27]. The enzymatic activity of 7α-dehydroxylation is known to only exist in limited number of intestinal microbiota belonging to genus Clostridium [28]. We found increased abundance of *Clostridium XlVa* and *Clostridium XVIII* in LD group. This may in turn lead to a higher level of 7α-dehydroxylase in intestinal and increase secondary bile acid levels, which are known to be related with higher biliary cholesterol secretion and favor gallstone formation. Berr et al. [29] have proved that increased activity of 7α-dehydroxylase expressed by gut microbiota was associated with the high levels of DCA in bile. High levels of DCA in gallbladder bile also correlated with fast cholesterol crystallization [30]. In contrast, inhibition of 7α- dehydroxylation activity of gut microbiota by antibiotics reducing DCA/CA ratio could lower cholesterol saturation of bile [31]. In contrast, the BSH activity exists in a broad spectrum of intestinal microbiota, which is common in *Bifidobacterium* and *Lactobacillus* [32]. LD tended to reduce *Lactobacillus*. Probiotics containing *Lactobacillus* had been shown to play a role in cholesterol-lowering properties both *in vivo* and *in vitro* [33–36]. They may suppress intestinal cholesterol absorption via assimilation of cholesterol, binding and incorporation of cholesterol into the cellular membrane, converting cholesterol into coprostanol and inhibit the formation of cholesterol micelles [37, 38].

The high level of cholesterol in lithogenic diet could increase intestinal permeability [39], which led to abnormal release of bacterial lipopolysaccharide (LPS) into plasma. Excessive amounts of LPS caused cholesterol accumulation and liver injuries via activation of inflammatory response. Antibiotic-induced inhibition of gut microbiota could aggravate all these disorders. High fat diet or “western diet” which contained high cholesterol only with no cholic acid increased in *Firmicutes* and a ratio of *Firmicutes* to *Bacteroidetes* in mice [40–43]. However, in the presence of cholic acid, we observed a profound decrease in *Firmicutes*. Since bile acids have strong antimicrobial activity [44], the discrepancies reflected the strong selective pressure on the gut microbiota by bile acids in modulation of the microbiota composition. Islam et al. [45] investigated the alterations in the gut microbiota after administration of cholic acid alone in rats. They found that feeding with a diet containing 0.5 g/kg or 2 g/kg cholic acid for 10 days could increase *Firmicutes* and decrease *Bacteroidetes*. While in our study, the gut microbiota profile was affected by both cholesterol and higher concentration of cholic acid (5 g/kg) for longer period (56 days). The difference response of gut microbiota in response to cholic acid might be due to the differences in dose and length of exposure. Moreover, it seemed that specie difference on gut microbiota in response to bile acids might also be present.

Lithogenic diet led to increase of the genera *Akkermansia*, a mucin-degrading bacterium. Previous studies suggested that *Akkermansia* could strengthen enterocyte monolayer integrity [46]. Subsequent study demonstrated that *Akkermansia* had the ability to fortify the impaired gut mucosal barrier after high fat diet, which alleviated metabolic endotoxemia caused by serum LPS [47]. Under such status, LD diet was expected to influence the gut epithelial integrity and the intestinal permeability due to the changes of *Akkermansia*.

## Conclusion

Our results showed dramatic alteration in abundance and composition of gut microbiota during the process of gallstone formation induced by lithogenic diet. Such changes in gut microbiota may contribute to the metabolic disorders of cholesterol and bile acid, which were significant factors contributing to the formation of cholesterol gallstone.

## Additional file

Additional file 1: The unique OTUs and their taxonomic profiles in chow and LD group. (DOC 58 kb)

## Abbreviations

ACE: Abundance-based cover-age estimator
BSH: Bile salt hydrolases
CA: Cholic acid
DCA: Deoxycholic acid
LD: Lithogenic diet
LDA: Linear discriminant analysis
LEfSe: LDA effect size
LPS: Lipopolysaccharide
PCoA: Principal coordinate analysis
RDP: Ribosomal Database Project
SPF: Specific pathogen free
