# Supplementary material for: Alteration of gut microbiota in association with cholesterol gallstone formation in mice
Source: BMC Gastroenterol. 2017 Jun 9;17:74. doi: 10.1186/s12876-017-0629-2 (PMC5466737; doi:10.1186/s12876-017-0629-2)
Supplement: Additional file 1: — The unique OTUs and their taxonomic profiles in chow and LD group. (DOC 58 kb) [file 12876_2017_629_MOESM1_ESM.doc]

**Table S1** **The unique OTUs and the corresponding taxonomic profiles in chow and LD group**

|  | **OUT ID** | **Phylum** | **Class** | **Order** | **family** | **genus** | **species** |
| --- | --- | --- | --- | --- | --- | --- | --- |
| **Chow** | OTU2 | *Bacteroidetes* | *Bacteroidia* | *Bacteroidales* | *Porphyromonadaceae* | *Barnesiella* | *Gram-negative_bacterium_cL10-2b-4* |
|  | OTU6 | *Firmicutes* | *Clostridia* | *Clostridiales* | *Lachnospiraceae* |  |  |
|  | OTU34 | *Proteobacteria* | *Deltaproteobacteria* | *Desulfovibrionales* | *Desulfovibrionaceae* | *Unclassified_Desulfovibrionaceae* | *uncultured_bacterium* |
|  | OTU35 | *Firmicutes* | *Clostridia* | *Clostridiales* | *Ruminococcaceae* |  |  |
|  | OTU96 |  |  |  |  |  |  |
|  | OTU97 | *Actinobacteria* | *Actinobacteria* | *Coriobacteriales* | *Coriobacteriaceae* |  |  |
|  | OTU103 |  |  |  |  |  |  |
|  | OTU139 | *Firmicutes* |  |  |  |  |  |
|  | OTU161 |  |  |  |  |  |  |
|  | OTU178 |  |  |  |  |  |  |
|  | OTU217 | *Firmicutes* | *Clostridia* | *Clostridiales* |  |  |  |
|  | OTU227 | *Firmicutes* | *Clostridia* | *Clostridiales* | *Ruminococcaceae* |  |  |
|  | OTU233 | *Firmicutes* | *Clostridia* | *Clostridiales* |  |  |  |
|  | OTU245 | *Proteobacteria* | *Alphaproteobacteria* |  |  |  |  |
|  | OTU261 | *Firmicutes* | *Clostridia* | *Clostridiales* | *Ruminococcaceae* |  |  |
|  | OTU267 | *Bacteroidetes* | *Bacteroidia* | *Bacteroidales* | *Porphyromonadaceae* | *Barnesiella* |  |
| **LD** | OTU50 |  |  |  |  |  |  |
|  | OTU51 |  |  |  |  |  |  |
|  | OTU65 |  |  |  |  |  |  |
|  | OTU99 |  |  |  |  |  |  |
|  | OTU154 | *Firmicutes* | *Clostridia* | *Clostridiales* | *Ruminococcaceae* | *Ruminococcus* |  |
|  | OTU171 | *Bacteroidetes* | *Bacteroidia* | *Bacteroidales* | *Rikenellaceae* | *Alistipes* |  |
|  | OTU275 | *Firmicutes* | *Clostridia* | *Clostridiales* | *Ruminococcaceae* |  |  |
|  | OTU288 | *Firmicutes* | *Clostridia* | *Clostridiales* | *Lachnospiraceae* |  |  |
|  | OTU302 |  |  |  |  |  |  |
